# Characterization of the Asiatic Acid Glucosyltransferase, UGT73AH1, Involved in Asiaticoside Biosynthesis in *Centella asiatica* (L.) Urban

**DOI:** 10.3390/ijms18122630

**Published:** 2017-12-06

**Authors:** Ok Tae Kim, Mei Lan Jin, Dae Young Lee, Reinhard Jetter

**Affiliations:** 1 Department of Herbal Research, National Institute of Horticultural and Herbal Science, RDA, Eumseong 27709, Korea; milan80623@korea.kr (M.L.J.); dylee0809@korea.kr (D.Y.L.); 2 Department of Botany, University of British Columbia, 6270 University Blvd., Vancouver, BC V6T 1Z4, Canada; Reinhard.jetter@botany.ubc.ca; 3 Department of Chemistry, University of British Columbia, 2036 Main Mall, Vancouver, BC V6T 1Z1, Canada

**Keywords:** *Centella asiatica*, transcriptome, triterpene saponins, UDP-glycosyltransferase

## Abstract

*Centella asiatica* (L.) Urban contains two ursane-type triterpene saponins, asiaticoside and madecassoside, as major secondary metabolites. In order to select candidate genes encoding UDP-glucosyltransferases (UGTs) involved in asiaticoside biosynthesis, we performed transcriptomic analysis of leaves elicited by methyl jasmonate (MeJA). Among the unigenes, 120 isotigs and 13 singletons of unique sequences were annotated as UGTs, including 37 putative full-length cDNAs, and 15 of the putative *UGT* genes were named according to the UGT committee nomenclature protocols. One of them, UGT73AH1, was characterized by heterologous expression in *Escherichia coli* BL21 (DE3) cells. After induction with IPTG, a total protein extract was assayed with UDP-glucose and asiatic acid. UPLC-QTOF/MS analysis showed that UGT73AH1 catalyzes the glycosylation of asiatic acid to its monoglucoside. It remains unclear whether glycosylation occurs on the triterpene C-2α, C-3β, C-23, or C-28 position. However, it is very likely that UGT73AH1 glucosylates the C-28 position, because only C-28 bears a glucose moiety in the final pathway product of asiatic acid, while C-2α, C-3β, and C-23 remain un-conjugated.

## 1. Introduction

Many plant secondary metabolites have characteristic carbon skeletons linked to one or more sugar moieties, and such glycosylation strongly affects the solubility, biochemical properties, subcellular localization, and biological function of the natural products [1]. Typically, glycosylation occurs only after the carbon skeletons of the aglycones have been established, in the final steps of the natural product biosynthesis pathways. Respective glycosylation reactions are generally catalyzed by transferases using activated sugar substrates, belonging to the enzyme family of UDP-glycosyltransferases (UGTs) [2].

Plants produce a large variety of triterpene saponins, with molecular structures featuring an alicyclic triterpene backbone linked through glucosidic bonds to one or more sugar moieties [3]. More than 300 different triterpenoid carbon skeletons have been described, and they can carry diverse glycosyl side chains on one or more oxygen functionalities [4,5,6]. Many plant species contain triterpene saponins derived from oleanane-type triterpenoids, such as glycyrrhizin from licorice, saikosaponins from *Bupleurum falcatum*, and soyasaponins from *Glycine max*. 

The functional characterization of *UGT* genes involved in triterpenoid biosynthesis has seen relatively little progress over the last decades, likely because all plant genomes encode numerous UGTs homologs, making it difficult to select and characterize candidate genes [7]. For example, the *Arabidopsis thaliana* and *G. max* genomes contain 115 and 242 *UGT* genes, respectively [8,9,10,11]. Recently, transcriptome sequencing has emerged as a tool to identify candidate *UGT* genes involved in the biosynthesis of particular triterpene saponins. In particular, the *UGT73K1* and *UGT71G1* genes from *Medicago truncatula*, *UGT73P2* and *UGT91H4* from *G. max*, *UGT74M1* from *Saponaria vaccaria*, *UGT73C10* and *UGT73C11* from *Barbarea vulgaris*, and *UGT74H6* from *Avena strigosa*, were shown to be involved in glycosylation of oleanane saponins [9,12,13,14,15,16]. Thus, enzymes belonging to the UGT subfamilies 71 to 74 appear able to link a glucose to the C-3β or C-28 functionalities of oleanane structures, implying that highly conserved motifs may convey some product specificity for triterpene glycosylation. However, only one gene, *UGT73AD1*, encoding a UGT responsible for forming ursane-type triterpene saponins has been identified to date [17], and it hence remains to be determined whether UGT71-74 homologs may also accept this different type of aglycone substrate.

The medicinal plant *Centella asiatica* (L.) Urban contains mainly ursane-type triterpene saponins, the most prominent ones being madecassoside and asiaticoside [18]. Both madecassoside and asiaticoside accumulate to relatively high concentrations in the leaves of *C. asiatica*, which have traditionally been used in various pharmaceutical applications. It is now firmly established that the major bioactivities of *C. asiatica* leaf extracts are due to these saponins, including memory improvement, wound and vein healing, antihistaminic, antiulcer and antilepsory treatments, as an antidepressant, and as antibacterial, antifungal, and antioxidant agents [19,20,21].

**Figure 1:**
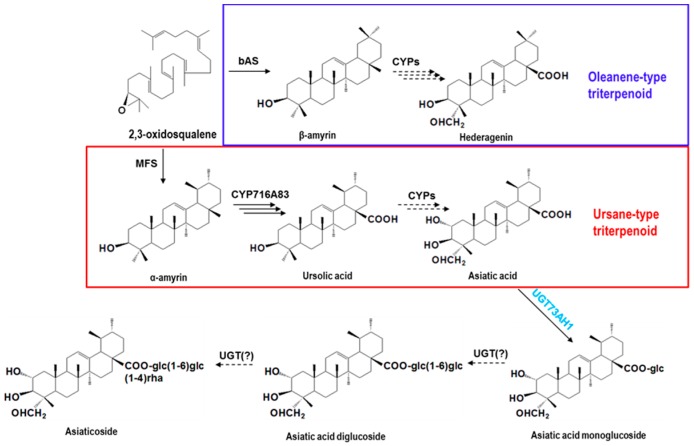
Putative biosynthetic pathway leading to triterpene saponins in *Centella asiatica*. The precursors, β-amyrin and α-amyrin, are converted by a series of oxidative reactions to oleanene- and ursane-type aglycones, respectively. In the ursane-type triterpenoid pathway, asiatic acid is converted by UDP-glucosyltransferases (UGTs) to asiaticoside. The following enzymes are represented: bAS, β-amyrin synthase; MFS, multifunctional triterpene synthase; CYP716A83, C-28 oxidase; CYPs, cytochrome P450 monooxygenases; UGT73AH1, 28-*O*-glucosytransferase.

Asiaticoside and madecassoside have identical sugar chains (glucose-glucose-rhamnose) bonded to their carboxyl groups [22]. Therefore, it has been hypothesized that both compounds are formed by glycosylation of asiatic acid and madecassic acid, respectively, probably catalyzed by UDP-glucosyltransferases (UGTs) that first link a glucose to the C-28 carboxyl group. A further glycosylation and a consecutive rhamnosylation reaction then likely lead to the final products (Figure 1). Recently, de Costa et al. [17] reported that UGT73AD1 plays a role in linking a glucose at a carboxyl moiety in *C. asiatica*. However, other *UGT* genes have not been characterized, to date.

Therefore, the goal of the present study was to isolate and characterize the glycosyl transferase(s) responsible for the formation of asiaticoside triterpene saponins in *C. asiatica*. To this end, we selected *UGT* candidate genes from transcriptomic data of elicited *C. asiatica* leaves, and characterized them by heterologous expression.

## 2. Results and Discussion

The goal of the present study was to identify one or more glycosyl transferases involved in asiaticoside formation using an omics approach. In particular, the transcriptome of MeJA-elicited *C. asiatica* leaves was analyzed, glycosyltransferase unigenes were selected based on phyologeny and expression patterns, and the resulting candidate genes were biochemically characterized using heterologous expression in *Escherichia coli* and product analysis with UPLC-QTOF/MS.

### 2.1. Transcriptome Analysis of Leaves Elicited with MeJA

Transcriptome analysis of elicited *C. asiatica* leaves yielded a total of 1,282,298 raw reads of 415.4 bps on average. After deleting adaptor sequences and eliminating reads < 100 bps, 40,064 unique sequences comprising 31,050 isotigs and 9014 singletons were identified and annotated, based on sequence similarity with complete sequences in the Swiss-Prot, TAIR (The Arabidopsis Information Resource), NCBI non-redundant protein (Nr), and NCBI non-redundant nucleotide (Nt) databases (Table 1). A total of 25,978 unigenes had significant matches in the databases. Overall, 64.8% of the unique sequences were annotated from all databases under highly stringent conditions (cutoff = 10^−3^).

### 2.2. Selection of Candidate UGT Genes for Asiaticoside Biosynthesis

The *C. asiatica* transcriptome dataset contained 133 unique sequences annotated as UGTs based on BLAST similarity, including 120 isotigs and 13 singletons, and 37 putative full-length cDNAs (Table S1). In comparison, Sangwan et al. [23] had reported 169 unique UGT sequences based on transcriptome analysis of *C. asiatica* leaves using Hiseq data, however, without providing information on gene names. Of the 37 UGTs identified here, 15 were named according to the UGT nomenclature rules [24], while the remaining 22 sequences could not be assigned as they lacked highly conserved motifs (possibly because they were truncated or incomplete). None of the sequences identified here had significant similarity with the *UGT73AD1* gene previously reported to be involved in oxidation of asiatic acid.

**Figure 2:**
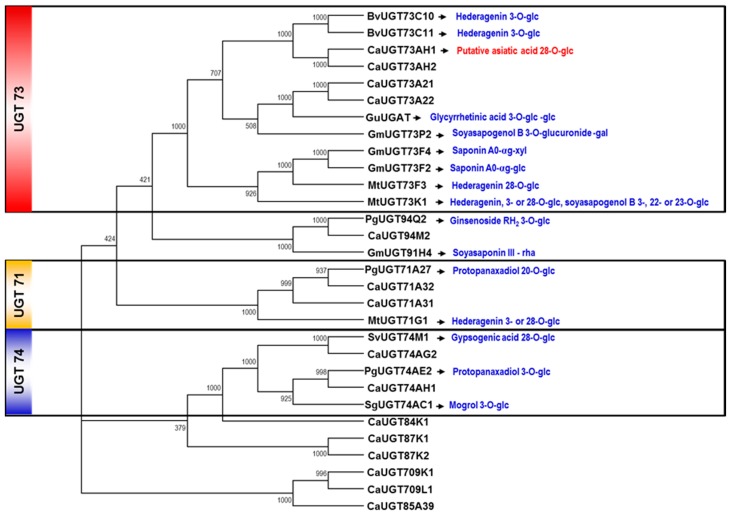
Phylogenetic analysis of UGTs from *C. asiatica* and other plant species. Sequences of UGTs associated with triterpene saponin biosynthesis were retrieved from the NCBI database: MtUGT71G1 (AAW56092), PgUGT71A27 (KM491309), BvUGT73C10 (AFN26666), BvUGT73C11 (AFN26667), MtUGT73K1 (AAW56091), MtUGT73F3 (ACT34898), GmUGT73F2 (BAM29362), GmUGT73F4 (29363), GmUGT73P2 (BAI99584), GuUGAT (KT759000), SvUGT74M1 (ABK76266), PgUGT74AE2 (JX898529), SvUGT91H4 (BAI99585), and PgUGT94Q2 (JX898530).

Six of the *UGT* candidate genes identified here belonged to the UGT73 and UGT74 subfamilies previously implicated in triterpene glycosylation. In particular, four of the *C. asiatica* UGTs (UGT73AH1, UGT73A21, UGT73A22, and UGT73AH2) belonged to the UGT73 subfamily, which comprises the *UGT73K1*, *UGT73P2*, and *UGT73C10* genes encoding triterpene glucosyltransferases of *M. truncatula*, *G. max*, and *Barbarea vulgaris*, respectively (Figure 2). On the other hand, two *C. asiatica* UGTs (*UGT74AH1* and *UGT74AG2*) belonged to the UGT74 family, which comprises the *UGT74M1* and *UGT74H6* genes known to be involved in saponin formation in *S. vaccaria* and *Avena strigosa*, respectively. All six *C. asiatica UGT73/74* genes had high EST representation and RPKM (Reads per Kilobase per Milion mapped reads) values (Table 2), and were therefore considered as good candidates to be involved in asiaticoside formation.

**Figure 3:**
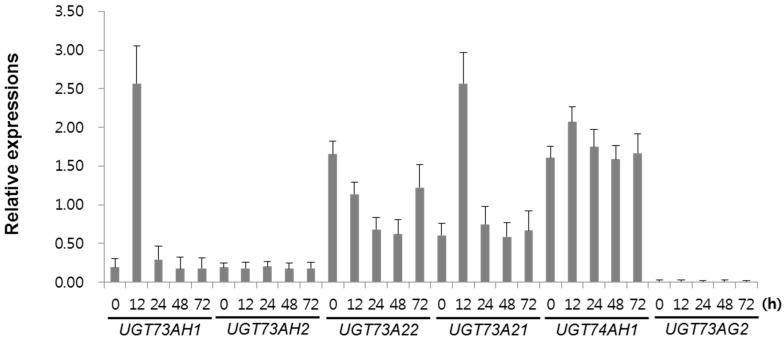
qRT-PCR analysis of UGT expression in hairy roots of *C. asiatica* after treatment with MeJA. The β-actin gene was used as a reference. The error bars represent standard errors from three biological replicates.

MeJA is known to strongly stimulate expression of *C. asiatica* genes encoding enzymes along the biosynthetic pathway from farnesyl diphosphate to triterpene glycosides. We therefore hypothesized that UGTs involved in asiaticoside and/or madecassoside formation should also be relatively highly expressed upon MeJA induction. Hence, in order to select strong candidates of UGT, we used qRT-PCR to compare the expression levels of the six *UGT* candidate genes in *C. asiatica* hairy root cultures elicited by 0.1 mM MeJA for 24 h (Figure 3). Transcripts of four *UGT* genes, *UGT73AH1*, *UGT73A21*, *UGT73A22*, and *UGT74AH1*, were upregulated after 12 h of MeJA treatment. In contrast, no significant differences were detected in the expression of the *UGT73AH2* gene in response to MeJA treatment, and transcripts of *UGT74AG2* could be detected neither in control nor in treated hairy roots. Overall, our qRT-PCR results indicated that four genes, *UGT73AH1*, *UGT73A21*, *UGT73A22*, and *UGT74AH1*, might be involved in asiaticoside biosynthesis.

### 2.3. Functional Characterization of Candidate UGTs

The deduced polypeptides of UGT73AH1, UGT73A21, UGT73A22, and UGT74AH1 consist of 501, 479, 471 and 462 amino acids, respectively, with amino acid identities between them ranging from 28% to 40% (Figure S1). All four *C. asiatica* UGTs possess the PSPG box, a highly conserved region among UGT enzymes associated with plant secondary metabolism [2]. 

**Figure 4:**
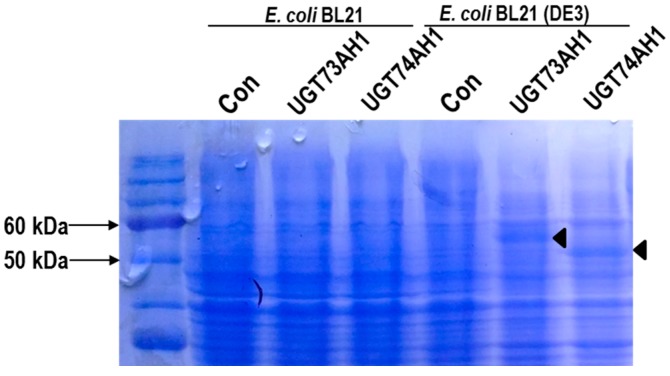
SDS-PAGE analysis of UGT73AH1 and UGT74AH1 heterologously expressed in *Escherichia coli.* Expression of the proteins in *E. coli* BL21 and BL21 (DE3) was induced by IPTG for 16 h at 20 °C.

To test the four candidate genes for UGT activity on triterpene substrates, heterologous expression in *E. coli* was used. Initially, the four candidate proteins were expressed with His-tags, and SDS-PAGE analysis showed that they accumulated to high levels upon induction (data not shown). However, the proteins were found mainly in inclusion bodies, and all four *UGT* genes therefore had to be re-cloned into vectors with stop codons to enhance their solubility [25]. The un-tagged proteins were expressed in two *E. coli* strains, BL21 and BL21 (DE3), however, no soluble protein products were observed for UGT73A21 and UGT73A22 (Figure 4). UGT73AH1 and UGT74AH1 were detected in cell-free extracts by SDS-PAGE analysis, and the two UGT activities were therefore examined further.

**Figure 5:**
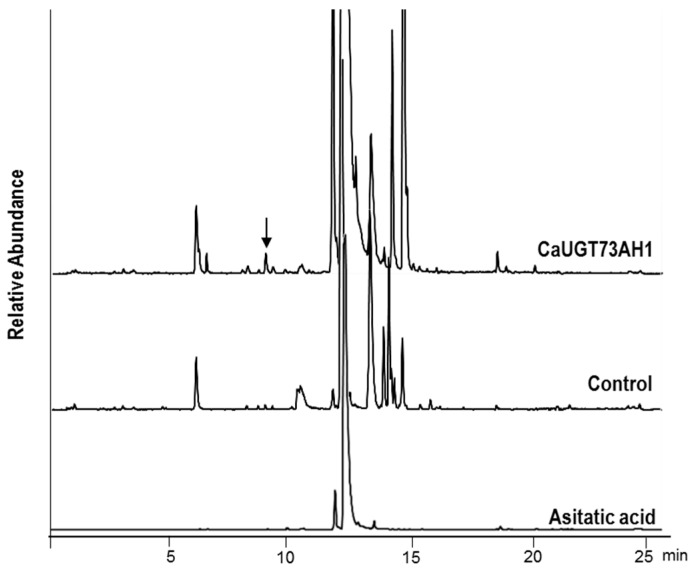
UPLC-QTOF/MS analysis of the products from glycosylation assays of UGT73AH1. Extracts from incubation mixtures containing the protein together with asiatic acid and UDP-glucose are compared with a boiled-protein control and authentic asiatic acid standard. The narrow indicates a peak in the protein incubation extract not present in the control and standard traces. The mass spectral identification of this peak is shown in Figure 6.

**Figure 6:**
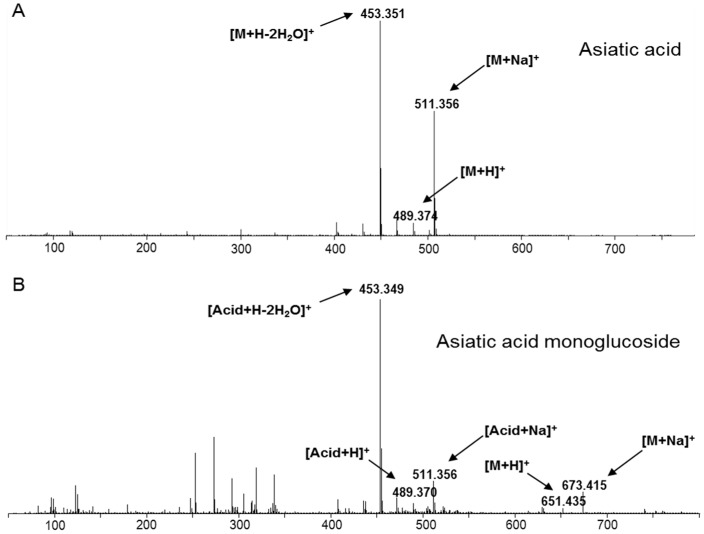
UPLC-QTOF/MS analysis of the products from glycosylation assays of UGT73AH1. (**A**) Mass spectrum of asiatic acid standard; (**B**) Mass spectrum of the unique compound formed from enzymatic reaction with UGT73AH1 protein. The fragments *m*/*z* 453, 489, and 511, identical to those of the standard, identify this compound as an asiatic acid derivative. The additional fragments *m*/*z* 651 and 673 further characterize it as a mono-glycoside.

For biochemical characterization, whole-cell extracts of *E. coli* expressing the candidate UGTs were assayed in vitro. After incubation of UDP-glucose and asiatic acid as substrates together with either UGT73A21 or UGT73A22 protein, the extracts were analyzed by UPLC-TOF/MS. In the extract from enzymatic mixture with UGT74AH1 protein only, compounds were detected that were also present in the control with denatured protein heated at 100 °C for 10 min, suggesting that this enzyme has no glycosylation activity on asiatic acid. In stark contrast, glycosylation assays with UGT73AH1 resulted in a UPLC peak eluting at 9.2 min under our conditions, which was not detected in the control sample (Figure 5). This compound was unambiguously identified by mass spectra as a mono-glycoside of asiatic acid, with *m*/*z* = 673.415 (Asiatic acid + Glc + Na)^+^ and *m*/*z* 651.453 (Asiatic acid + Glc + H)^+^ , reflecting the presence of both a hexose moiety and asiatic acid (Figure 6). Taken together, our results show that UGT73AH1 catalyzes the glycosylation of asiatic acid.

Asiatic acid has hydroxyl groups at C-2α, C-3β, and C-23, as well as a carboxyl moiety at C-28, and thus, four potential glycosylation sites. Unfortunately, our mass spectrometric and chromatographic data alone do not reveal the exact position of the glucose unit in the enzyme assay product. Instead, NMR analysis would have to be performed to elucidate the exact structure of the peak, however, limited sample amounts impeded further analysis so far. It could therefore not be assessed whether UGT73AH1 attaches the hexose to one of the hydroxyls or the carboxyl of asiatic acid, and the (product) regio-specificity of the enzymes remains to be determined. 

To date, 33 oleanane- and ursane-type saponins have been reported in *C. asiatica* [26], all of them containing a glucose linked to the triterpene C-28 carboxyl, whereas no glucose linked with C-2α, C-3β, and C-23 positions has been observed. Therefore, it seems very likely that UGT73AH1 catalyzes glycosylation at the C-28 carboxyl function en route to the asiaticoside triterpene saponins.

## 3. Materials and Methods

### 3.1. Plant Material and Sampling

Whole plant cultures of *C. asiatica* were established from node segments as previously described by Kim et al. [18]. Plants generated from four independent node segments were cultured on 1/2 Murashige and Skoog (MS) liquid medium supplemented with 3% sucrose at 23 ± 2 °C under light at 100 rpm for 2 weeks, then 0.1 mM methyl jasmonate (MeJA) (Sigma-Aldrich, Darmstadt, Germany) was added to the medium. Twenty-four hours after MeJA elicitation, leaves were harvested, immediately frozen in liquid nitrogen, and used for extraction of total RNA.

### 3.2. EST Assembly and Annotation

Total RNA samples of *C. asiatica* were sequenced using the Genome sequencer FLX (454 Life Sciences, Roche, Basel, Switzerland), and the raw data were processed with the Roche GS FLX software (version 2.5.3, 454 Life Sciences, Roche, Basel, Switzerland) at quality score threshold 40 and quality-trimmed with Lucy software (version 2.19; available online: http://lucy.sourceforge.net/) [27] and SeqClean (version 1.0; available online: http://compbio.dfci.harvard.edu). The resulting sequence reads were assembled de novo using the software package Newbler (a) set to extend low-depth overlaps. The assembled unique transcripts were first compared to the GenBank database using the BLASTN algorithm with an *E* value cut-off of 10^−3^, to identify and remove ribosomal RNA sequences. The remaining sequences, putatively encoding proteins, were searched against the *Arabidopsis* protein database at The Arabidopsis Information Resource (TAIR; available online: http://www.arabidopsis.org), the Swiss-Prot protein database (Available online: http://www.expasy.ch/sprot; released on 11/20/2013), and the NCBI non-redundant protein (Nr) database (Available online: http://www.ncbi.nlm.nih.gov; released on 11/20/2013) using the BLASTX algorithm with an *E* value cut-off of 10^−3^. All the resulting *C. asiatica* high-quality reads were deposited at NCBI, and can be accessed in the Sequence Read Achieve (SRA) database under project accession number SRX1050304.

### 3.3. Expression Levels of UGT Genes in C. asiatica

Expression levels of all isotigs were calculated using the RPKM (Reads Per Kilobase per Milion mapped reads) method [28], with the formula RPKM(A) = (1,000,000 × C)/(N × L × 1000), where RPKM(A) is the expression of gene A, C is the number of reads that uniquely align to gene A, and N is the total number of bases in gene A, and L is the exon length in base pairs. Statistical comparisons were performed as described by Audic and Claverie [29]. FDR (False Discovery Rate) correction was used to determine the *p* values in multiple tests. Unigenes were considered differentially expressed when the RPKM of treated sample and control displayed a more than twofold change, with *p* less than 10^−3^.

### 3.4. Phylogenetic Tree Analysis

The protein sequences were aligned with the CLUSTALW program and phylogenetic trees constructed using the MEGA5.2 software [30] using the neighbor-joining method and bootstrapping for 1000 replicates. 

### 3.5. Real-Time RT-PCR Analysis

Hairy roots of *C. asiatica* treated with MeJA for 0, 12, 24, 48, or 72 h cDNAs were used to harvest total RNA as described by Kim et al. [31]. First-strand cDNA was synthesized using the AMV reverse transcriptase (Promega, Medison, WI, USA) and 2 µg of total RNA and SYBR Green Master Mix (Bio-Rad, Hercules, CA, USA) for quantification with the CFX96TM Real-Time System (Bio-Rad) and gene-specific primers (Table S2). PCR conditions were as follows: initial denaturation at 95 °C for 3 min, followed by 40 cycles of amplification for 15 s at 95 °C, for 15 s at 58 °C and for 30 s at 72 °C. After completing the reactions, the threshold cycle (*C*_t_) value for each reaction was obtained, and the differences were calculated using the delta-delta-*C*_t_ method, with the β-actin gene (GenBank accession No. JK517508) as internal control. The fold change in transcript levels for each gene is presented as the mean and standard error of three independent experiments.

### 3.6. Expression of UGT Genes

Full-length cDNAs of target UGT were cloned using the Gateway system with specific primers, based on sequences from the transcriptome data analysis. Sequence information for the putative UGT genes has been deposited in GenBank with accession numbers (UGT73AH1, MF471454; UGT73A21, MF471455; UGT73A22, MF471459; UGT74AH1, MF471458). To minimize inclusion bodies of recombinant proteins, complete open reading frames (ORFs) with stop codon were used [25]. The ORFs were amplified by PCR from the original cDNA with attB-modified custom primers for four UGTs, and the PCR products were inserted into the pDONR/Zeo entry vector following the manufacturer’s protocol. Plasmid DNAs were prepared from several transformants and sequenced. The *E. coli* expression clone was constructed using the entry vector and a destination vector pET62-DEST (Merck KGaA, Darmstadt, Germany) following the protocol provided. After confirmation of sequences, the plasmids were used for *E. coli* transformation.

Single colonies of BL21 (DE3) cells harboring the pET-UGT73AH1 or pET-UGT74AH1 vectors were cultured at 37 °C in liquid lysogeny broth (LB) medium containing 50 µg/mL ampicillin to an optical density of 0.5 at 600 nm. Then, the cultures were shifted to 28 °C, and isopropyl thio-β-d-galactoside (IPTG) was added to a final concentration of 0.5 mM, and incubation was continued at 20 °C for 16 h. Finally, cells were collected by centrifugation at 15,000× *g* for 5 min, suspended in extraction buffer (50 mM sodium phosphate, pH 7.4, 300 mM sodium chloride, 20% glycerol), and then disrupted by sonication for three times 10 s. After cell debris was removed by centrifugation at 15,000× *g*, the supernatant was checked for the presence of the expressed protein using 10% SDS-PAGE. The total protein extract was used for in vitro assays, performed by incubating 1 mg protein in 1.5 mL 50 mM Tris-HCl, pH 8.5, containing 5 mM uridine-5′-diphosphoglucose and 0.5 mM asiatic acid, at 35 °C for 4 h. Finally, 1.5 mL butanol was added to stop the reaction and extract products, and the organic layer was collected, and the solvent evaporated under nitrogen gas. The resulting residue was re-dissolved in 200 µL of methanol, and stored at 4 °C for chemical analysis.

### 3.7. UPLC-QTOF/MS Analysis of the Products from Enzymatic Reactions

UPLC was performed using an ACQUITY H-Class UPLC (Waters, Milford, MA, USA) according to the protocol reported by Lee et al. [32] with an ACQUITY BEH C18 column (2.1 mm × 100 mm, 1.7 µm) held at 40 °C. Two microliters of sample were injected for separation, with a mobile phase consisting of Solvent A (5% acetonitrile, 0.1% formic acid) and Solvent B (95% acetonitrile, 0.1% formic acid) at a constant flow rate of 450 µL/min. Eluting compounds were detected with a Waters Xevo G2-S QTOF-MS (Waters) operating in positive and negative ion mode, with alternating high- and low-energy scans (MSE acquisition mode) and cone voltage 40 V, capillary voltage 3.0 kV, source temperature 120 °C, desolvation temperature 300 °C, cone gas flow 30 L/h, and desolvation gas flow 600 L/h. Accurate mass measurements were obtained by means of an automated calibration delivery system using leucine as internal reference (*m*/*z* 556.276 (ESI^+^), *m*/*z* 554.262 (ESI^−^)). Data were collected between *m*/*z* 100 and *m*/*z* 2000. 

## 4. Conclusions

Four candidate genes of UGT were selected from transcriptome data based sequence comparisons, and based on their expression patterns in MeJA-elicited *C. asiatica* leaves. UPLC-TOF/MS analysis of in vitro assays showed that UGT73AH1 produces asiatic acid monohexoside. We thus demonstrated that the UGT73AH1 protein catalyzes the glycosylation of a ursane-type triterpene, now enabling further investigations into the genetic engineering of triterpene saponin biosynthesis. As part of these studies, it will be interesting to test the relative activity of the enzyme on other substrates, including madecassic acid as well as oleanane backbone isomers of both asiatic and madecassic acids.

## Figures and Tables

**Table 1:** Summary of the annotation rates of *C. asiatica* unique sequences in various public database.

The Public Database	No. of Unique Sequences	Annotation Percentage (%)
TAIR	14,051	35.0
Swiss-Prot	15,171	37.9
Nr	17,807	44.4
Nt	16,709	41.7
Total	25,978	64.8

TAIR = The Arabidopsis Information Resource; Nr = NCBI non-redundant protein; Nt = NCBI non-redundant nucleotide.

**Table 2:** Selected *UGT* (UDP-glucosyltransferase) gene candidates resulting from transcriptome analysis of MeJA-elicited *C. asiatica* leaves.

Gene Name	Unique Sequence	EST Number	RPKM	Putative Function and Source	*E*-Value
*UGT73AH1*	Isotig09551	235	118.01	UDP-glycosyltransferase superfamily protein, putative (*Theobroma cacao*)	0
*UGT73A21*	Isotig08753	232	101.59	Glycosyltransferase UGT7 (*Bupleurum chinense*)	0
*UGT74AH1*	Isotig09014	130	59.50	UDP-glycosyltransferase 74E2-like (*Vitis vinifera*)	0
*UGT73A22*	Isotig09265	115	55.09	UDP-glycosyltransferase UGT7 (*Bupleurum chinense*)	0
*UGT74AG2*	Isotig08223	136	53.51	PREDICTED: UDP-glycosyltransferase 74E1-like (Solanum lycopersicum)	0
*UGT73AH2*	Isotig08281	72	28.72	UDP-glycosyltransferase 73C2 (*Vitis vinifera*)	0

EST = Expressed sequence tag; RPKM = Reads per kilobase per million mapped reads; UDP = Uridine-5’-diphosphate.

## Supplementary Materials

Supplementary materials can be found at www.mdpi.com/1422-0067/18/12/2630/s1.

## Abbreviations

UGT	UDP-glucosyltransferase
MeJA	Methyl jasmonate
RPKM	Reads per kilobase per million mapped reads
EST	Expressed sequence tag
UPLC-QTOP/MS	Ultra-high Performance Liquid Chromatography-Quadrupole Time-of-Flight Mass Spectrometry
