# Supplementary material for: Characterization of the Asiatic Acid Glucosyltransferase, UGT73AH1, Involved in Asiaticoside Biosynthesis in Centella asiatica (L.) Urban
Source: Int J Mol Sci. 2017 Dec 6;18(12):2630. doi: 10.3390/ijms18122630 (PMC5751233; doi:10.3390/ijms18122630)
Supplement: Supplementary file 1 [file ijms-18-02630-s001.zip › Figure S1. Alignment of the deduced amino acid sequences of four candidate genes.docx]

**
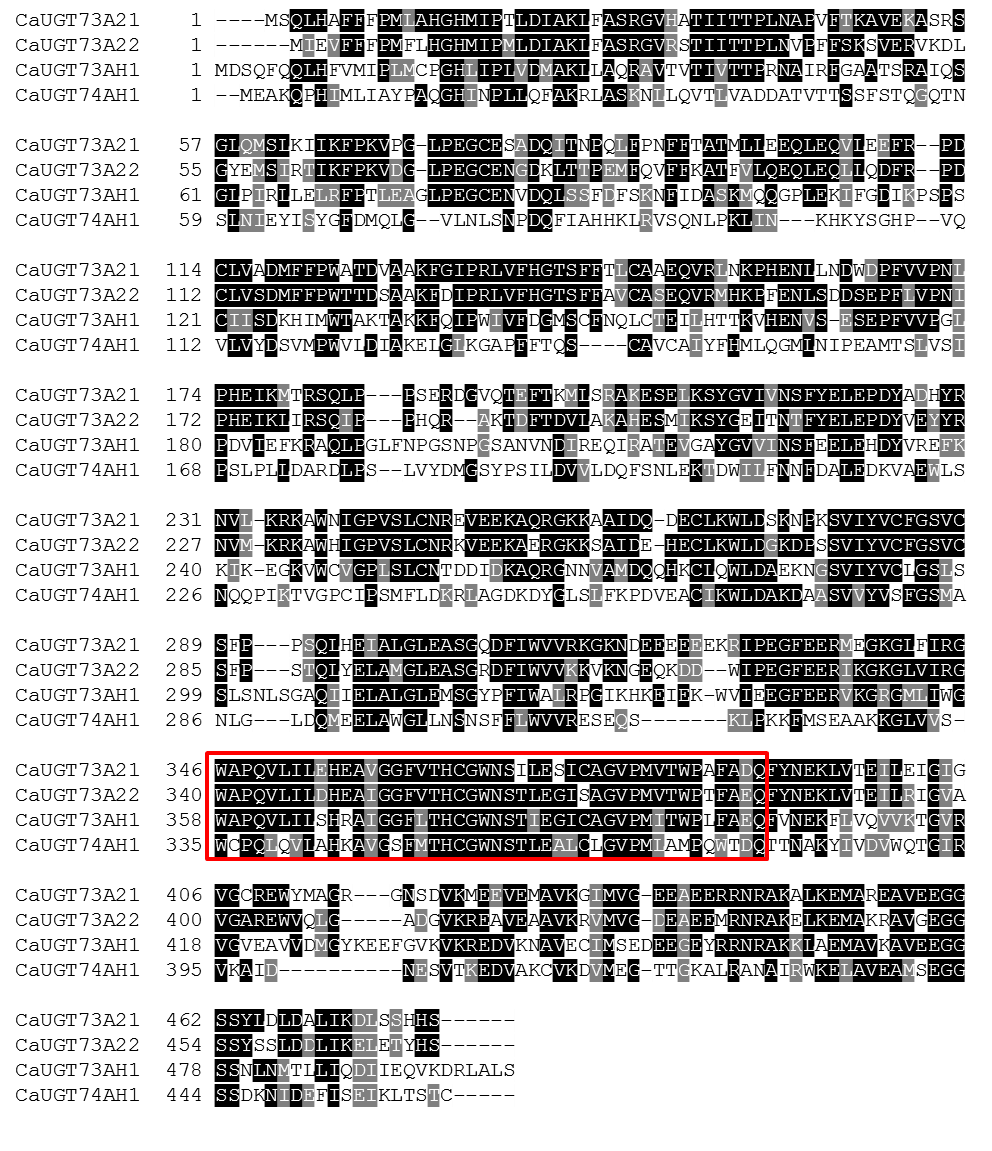
**

Figure S1. Alignment of the deduced amino acid sequences of four *C. asiatica* candidate UGT genes. Fully conserved residues are shaded in black. Grey shading indicates a similarity significance value of at least 70%. The PSPG box motif is marked by a frame
