# Supplementary material for: Characterization of the Asiatic Acid Glucosyltransferase, UGT73AH1, Involved in Asiaticoside Biosynthesis in Centella asiatica (L.) Urban
Source: Int J Mol Sci. 2017 Dec 6;18(12):2630. doi: 10.3390/ijms18122630 (PMC5751233; doi:10.3390/ijms18122630)
Supplement: Supplementary file 1 [file ijms-18-02630-s001.zip › Table S2. Oligonucleotide primers used in this study.docx]

Supplemental Table S2. Oligonucleotide primers used in this study

| Primers | | Sequence (5’ to 3’) |
| --- | --- | --- |
| Gateway subcloning | UGT73AH1-F  UGT73AH1-R  UGT73A21-F  UGT73A21-R  UGT73A22-F  UGT73A22-R  UGT74AH1-F  UGT74AH1-R | AAAAAGCAGGCTTCATGGATTCACAATTTCAGCAG  AGAAAGCTGGGTCGCTTAATGCTAACCTATCCTTTACT  AAAAAGCAGGCTTCATGAGTCAGCTTCATGCCTTCTTT  AGAAAGCTGGGTCGGAATGATGAGAACTCAAATCCTT  AAAAAGCAGGCTTCATGGAGTTGCTTTCCTCTTAT  AGAAAGCTGGGTCAGAATGATAAGTTTCCAATTCTTT  AAAAAGCAGGCTTCATGGAGGCAAAGCAACCTCAC  AGAAAGCTGGGTCACAAGTGCTAGTCAATTTAATTTCA |
| qRT-PCR |  |  |
|  | UGT73AH1-F  UGT73AH1-R  UGT73AH2-F  UGT73AH2-R  UGT73A21-F  UGT73A21-R  UGT73A22-F  UGT73A22-R  UGT74AH1-F  UGT74AH1-R  UGT74AG2-F  UGT74AG2-R  Actin-F  Actin-R | GCATCATGTCTGAAGATGAGG  GCTTAATGCTAACCTATCCTT  GCTTAATGTTTGAAGGGGAAG  TTACGCAGTGTTCATACTTAG  GGCAGTGAAGGGGATAATGGTG  GGAATGATGAGAACTCAAATC  GGCGGCAGTGAAGCGGGTAAT AGAATGATAAGTTTCCAATTC  GTGTGTGAAAGATGTCATGGA  ACAAGTGCTAGTCAATTTAA  CGCTCAGCTAGCTCCCTAAAC  GGTACAAGTGCTGAAACAGATTTT  GATGACATGGAAAAGATTTGGCATC  AAGGATGGCATGAGGGAGGGCGTAA |
